# Characterization of a Small Plaque Variant Derived from Genotype V Japanese Encephalitis Virus Clinical Isolate K15P38

**DOI:** 10.4014/jmb.2404.04054

**Published:** 2024-06-23

**Authors:** Woo-Jin Kim, Ah-Ra Lee, Su-Yeon Hong, Sang-Hyun Kim, Jae-Deog Kim, Sung Jae Kim, Jae Sang Oh, Sang-Mu Shim, Sang-Uk Seo

**Affiliations:** 1 Department of Biomedicine & Health Sciences, Graduate School, The Catholic University of Korea, Seoul 06591, Republic of Korea; 2 Department of Microbiology, College of Medicine, The Catholic University of Korea, Seoul 06591, Republic of Korea; 3 Vaxdigm Co., Ltd., Seoul 04798, Republic of Korea; 4 Bio & Living Engineering Major, Global Leaders College, Yonsei University, Seoul 03722, Republic of Korea; 5 Department of Neurosurgery, Uijeongbu St. Mary’s Hospital, College of Medicine, The Catholic University of Korea, Seoul 06591, Republic of Korea; 6 Division of Acute Viral Disease, Center for Emerging Virus Research, National Institute of Infectious Diseases, Korea National Institute of Health, Cheongju 28159, Republic of Korea

**Keywords:** Japanese encephalitis virus, genotype V, mutant virus, attenuated variant, small plaque variant

## Abstract

Genotype V (GV) Japanese encephalitis virus (JEV) has been predominantly reported in the Republic of Korea (ROK) since 2010. GV JEV exhibits higher virulence and distinct antigenicity compared to other genotypes, which results in reduced efficacy of existing vaccines. Research on GV JEV is essential to minimize its clinical impact, but the only available clinical strain in the ROK is K15P38, isolated from the cerebrospinal fluid of a patient in 2015. We obtained this virus from National Culture Collection for Pathogens (NCCP) and isolated a variant forming small plaques during our research. We identified that this variant has one amino acid substitution each in the PrM and NS5 proteins compared to the reported K15P38. Additionally, we confirmed that this virus exhibits delayed propagation in vitro and an attenuated phenotype in mice. The isolation of this variant is a critical reference for researchers intending to study K15P38 obtained from NCCP, and the mutations in the small plaque-forming virus are expected to be useful for studying the pathology of GV JEV.

## Introduction

The Japanese encephalitis virus (JEV), a member of the *Flaviviridae* family, is a positive-sense single-stranded RNA virus that is endemic to parts of temperate and tropical regions [1, 2]. Approximately 67,900 cases of Japanese encephalitis (JE) are reported annually in these endemic areas [2]. JE is a zoonotic disease transmitted by *Culex* mosquitoes, and various domestic and wild animals serve as amplifying hosts [3]. RNA viruses generally exhibit a higher mutation rate, allowing JEV to evolve into various variants and accumulate additional mutations during circulation within zoonotic hosts [4]. As a consequence, JEV has diverged and can be classified into five genotypes (GI to GV) based on the Envelope (E) gene sequence [5]. In earlier studies, GIII was the dominant strain of JEV, but since the mid-1990s, a clear prevalence of GI has been observed, and more recently, GIV and GV strains of JEV have been detected in some regions [6-9].

JE is preventable through vaccination and is effectively controlled in the Republic of Korea (ROK) through a nationwide vaccination program [10]. Since 2010, GV JEV has been continuously reported in the ROK, and the number of JE cases has concurrently increased [11]. Increased JE cases may be ascribed to inefficient vaccine efficacy, as some studies have shown that current commercially available JE vaccines are based on GIII and have weaker protective efficacy against GV compared to JEVs in other genotypes [12-14]. We have also reported that it is necessary to develop vaccines based on GV antigens to control infections by GV JEV more efficiently in animal models [15]. Although GV currently seems to be contained within the ROK, its previous detection in Malaysia and China suggests the possibility of a surge in other regions [16, 17]. Therefore, in addition to developing effective vaccines, it is essential to research the virological characteristics of GV strains to effectively respond their potential expansion.

To date, only two GV JEV isolates have been obtained from cerebrospinal fluid samples of JE patients: Muar, isolated in Singapore from Malaysian patients in 1952, and K15P38, isolated in the ROK in 2015 [8, 18]. Research on Muar has revealed that this virus exhibits higher virulence in mice compared to JEV strains belonging to other genotypes [17], suggesting a higher risk posed by GV strains to the human population. To analyze the characteristics of another GV JEV clinical isolate, K15P38, we obtained the isolate from the National Culture Collection for Pathogens (NCCP) under the code NCCP 43279 [19]. During the process of establishing a working bank for this virus, we were able to isolate a variant with smaller plaque size. From the point of patient infection to the creation of our working stock, there are numerous steps during which the variant could have emerged. However, it is difficult to pinpoint the exact moment of its origin. Nevertheless, considering the uniqueness and rarity of the GV clinical isolate, it is essential to characterize this small plaque variant.

## Materials and Methods

### Virus and Cells

The JEV isolate designated K15P38 utilized in this research was acquired from the NCCP with the assigned code of 43279. The Baby hamster kidney (BHK)-21 (C-13) cell line was procured from the Korean Cell Line Bank (KCLB). This cell line was cultured in Dulbeccós Modified Eagle Medium (DMEM) (HyClone, USA) supplemented with 10% fetal bovine serum (FBS) (HyClone) and 1× PS solution (Lonza, Switzerland). The culture conditions were maintained at 37°C in a 5% CO_2_ atmosphere. Viral stocks employed in the study were propagated at a multiplicity of infection (MOI) of 0.01 by infecting BHK-21 cells.

### Plaque Assay

BHK-21 cells were seeded at a 3.0 × 10^5^ cells per well in a 6-well plate and incubated at 37°C in a 5% CO_2_ atmosphere for 20 h prior to infection. The samples were serially diluted 10-fold in infection media composed of 2% FBS in DMEM, and were incubated with the cell monolayers for 1.5 h at 37°C in a 5% CO_2_ incubator. Following incubation, the monolayers were washed with DPBS (HyClone) and overlaid with 3 ml of media containing 2%SeaPlaque agarose (Lonza), 8% FBS, and 1× PS solution in Eagle’s Minimum Essential Medium (EMEM)(Welgene, Republic of Korea). After four days of incubation, the cells were fixed using 3 ml of 10% formalin (Biosesang, Republic of Korea) for two hours. The wells were then stained with a 1% crystal violet solution in 20%ethyl alcohol and thoroughly rinsed with tap water.

### Isolation of Small Plaque Virus

The monolayer of BHK-21 cells in a 6-well plate was infected with 30 plaque-forming units (pfu) of NCCP 43279 to generate plaques as described previously. After four days of infection, cytopathic effects (CPE) were observed under a microscope, and plaques with small diameters were identified for isolation. Individual plaques were carefully picked using sterile pipette tips, following the removal of the overlay media via suction. These plaques were then used to inoculate fresh monolayers of BHK-21 cells. The isolated virus was propagated in BHK-21 cells through two additional passages, with successful isolation confirmed by the formation of homogeneous small plaques.

### Sequencing of Viral Genome

Viral RNA was extracted from 200 μl of viral stock solution using the AccuPrep Viral RNA Extraction Kit (Bioneer, Republic of Korea). Complementary DNAs were synthesized and amplified employing the AccuPower RT-PCR Master Mix (Bioneer), following the manufacturer’s instructions with minor modifications. Sequencing of the amplified PCR products was performed in both directions (Cosmogenetech, Republic of Korea). The complete sequences were registered in GenBank under the accession numbers PP478074 for the large plaque isolate and PP582382 for the small plaque isolate.

### In Vitro Virus Growth and Cytotoxicity

A monolayer of BHK-21 cells in a 6-well plate was infected with a 0.01 MOI of JEV in infection media for 1.5 h. Subsequently, the wells were washed with DPBS (HyClone) and maintained in 2 ml of infection media. At various time points, supernatants were collected for viral titer assessment. Additionally, the number of adherent cells remaining in each well was counted to evaluate cytotoxicity.

### In Vivo Virus Characterization

Five-week-old female BALB/c mice were purchased from Orient Bio (Republic of Korea). Mice were intravenously infected with 10^6^ pfu of JEV and then randomly divided into two groups. One group was sacrificed four days post-infection and transcardially perfused with 20 ml of cold PBS prior to harvesting the brain tissue. The second group was monitored to assess lethality for 15 days post-infection. The humane endpoint was set at a body weight reduction of 20% or more compared to the initial weight. All mice were anesthetized with isoflurane and euthanized by CO_2_ inhalation. All animal research was conducted under protocols approved by the Institutional Animal Care and Use Committee at The Catholic University of Korea (approval no. CUMS-2023-0264-01).

### Histology

Mouse brains were carefully extracted and fixed in 10% formalin. Tissues were sliced into 5 μm sections and then stained with hematoxylin and eosin (H&E). All specimens were digitized using the Pannoramic MIDI Scanner (3DHistech, Hungary). A histological damage scoring system, adapted from a previous study, ranging from 0 (no damage) to 4 (extreme damage) was used to quantify the severity of the changes in the brain tissue.

### Flow Cytometry

The brain tissue was finely minced using a clean blade and incubated with a digestion buffer consisting of 1 mg/ml collagenase type 4 (Worthington Biochemical Corp., USA) and 50 μg/ml DNase I (Roche) in (HyClone) for 1 h at 37°C on a shaker. The samples were then sieved through a 70-μm nylon mesh strainer and centrifuged to collect the pellet. For density gradient centrifugation, the pellet was resuspended in 30% Percoll in PBS and layered into a centrifuge tube containing 37% Percoll overlaid on 70% Percoll. Following centrifugation, mononuclear cells were isolated from the interphase. A total of 1 × 10^5^ mononuclear cells were stained for 20 min at 4°C in FACS buffer comprising 2% FBS and 1 mM EDTA in PBS, using combinations of the following antibodies: CD45 (30-F11), CD11b (M1/70), and Ly6G (1A8) from BioLegend (USA); Ly6C (AL-21) from BD Biosciences (USA). The cells were then washed and resuspended in cold FACS buffer. Data were analyzed using the BD FACSCanto system and FlowJo v10.8.1 software (BD Biosciences).

### Cytokine Measurement

The brain was homogenized using the FastPrep-24 5G bead beating system (MP Biomedicals, USA) along with the homogenization kit (IGT-25ZS, InnoGeneTech, Republic of Korea) in 1 ml of PBS. The supernatants were collected and quantified using the Cytometric Bead Array Mouse Inflammation Kit (BD Biosciences) with a BD FACSCanto flow cytometer. Data were analyzed with the FCAP Array Software v3.0 (BD Biosciences).

### Statistical Analysis

Values represent the mean and standard deviation (SD) for all experiments. All data were analyzed using GraphPad Prism v9.5 (GraphPad, USA). Statistical analyses were performed using Student's *t*-test (two-tailed), analysis of variance (ANOVA), and the Kaplan-Meier method. Statistical significance was determined at a P-value of less than 0.05, denoted as follows: *p < 0.05; **p < 0.01; ***p < 0.001; ****p < 0.0001.

## Results

### Isolation of Small Size Plaque-Forming Virus

**Fig. 1:**
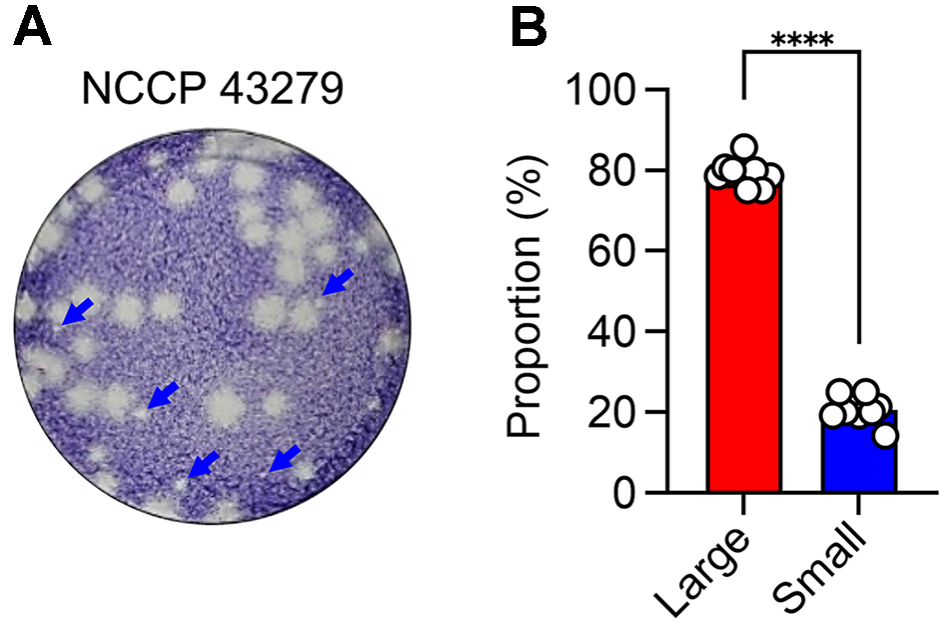
Identification of small plaque-forming K15P38 variant. (**A**) Representative plaque morphology of the NCCP 43279 strain. Small plaques were observed and are indicated by blue arrows. (**B**) The proportion of large and small plaques was measured from 10 individual wells, with approximately 50 plaques observed per well. Data are shown as mean ± SD values. ****p < 0.0001.

While establishing the NCCP 43279 master bank, we observed plaques of smaller size during the quantification of viral titers (Fig. 1A). We repeated a plaque assay across multiple wells and found that the proportion of small-sized plaques was 21.05 ± 3.19% among all plaques (Fig. 1B). We isolated viruses from plaques of different sizes, and for this study, we have designated each isolate based on plaque size as either 'Largé or 'Small'.

### Sequencing of Isolated K15P38 Viruses

**Fig. 2:**
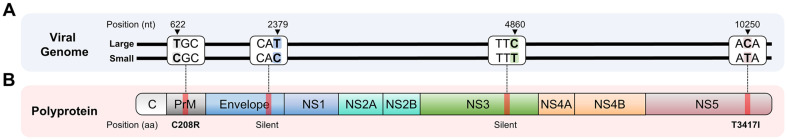
Gene mutations and amino acid substitutions of the small plaque-forming variant. (**A**) Location of four mutations on the viral genome. Mutations are indicated with black arrowheads at specific position numbers. Position numbers are counted based only on the open reading frame (ORF), excluding the untranslated regions. (**B**) Positions of amino acids on the translated polyprotein corresponding to the genetic mutations. Locations where substitutions occurred are highlighted in bold, with the substituted amino acids shown. nt: nucleotide, aa: amino acid.

We sequenced the nucleotide sequence of the entire open reading frame (ORF) of the Large and Small viruses. Differences in the sequences were identified at positions 622, 2379, 4860, and 10250 of the complete ORF, corresponding to the genes coding for PrM, E, NS3, and NS5, respectively (Fig. 2A). Among these, the T-to-C mutation at position 622 and the C-to-T mutation at position 10250 resulted in C208R and T3418I substitutions in the polyprotein, respectively (Fig. 2B).

### In Vitro Characterization of K15P38 Variants

**Fig. 3:**
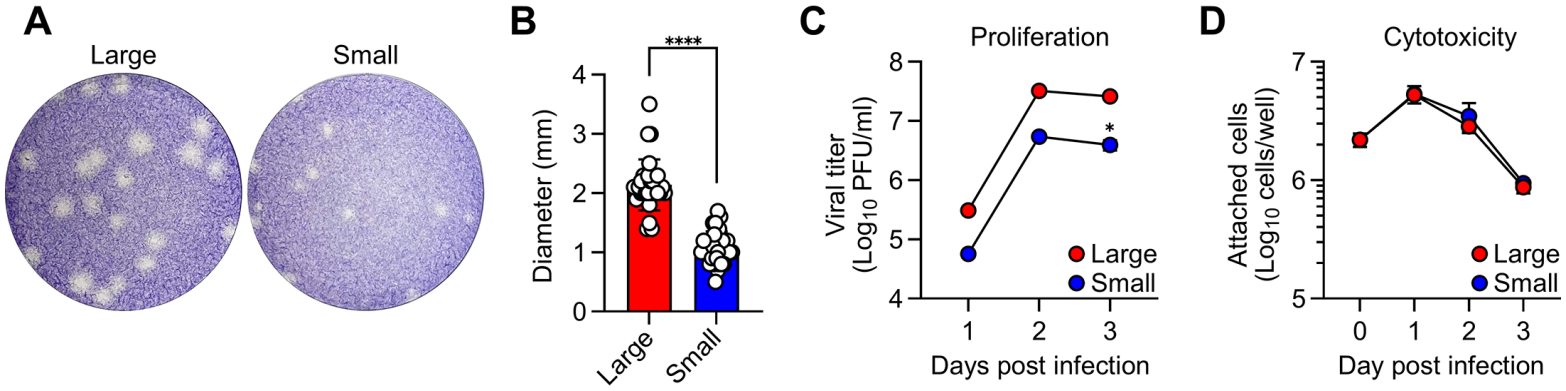
In vitro characterization of K15P38 variants. (**A**) Representative plaque images for Large and Small viruses. BHK-21 cells were infected with either virus, and images were captured 80 hours post-infection. (**B**) The diameter of thirty individual plaques from each virus type was measured in millimeters. (**C, D**) BHK-21 cells were infected with either the Large or Small virus at an MOI of 0.01. Viral titer (**C**) and the number of adherent cells (**D**) were assessed to examine the growth kinetics and cytotoxicity, respectively. Data represent mean ± SD values pooled from two independent experiments in duplicate wells (*n* = 4). *p < 0.05, ****p < 0.0001.

Plaque assays using isolated Large and Small isolates revealed generally homogenous plaques of different sizes (Fig. 3A). The plaque sizes were 2.14 ± 0.43 mm for Large and 1.08 ± 0.28 mm for Small (Fig. 3B). When proliferated in vitro, Small isolates showed slower propagation with a lower peak titer (5.5 ± 3.5 × 10^6^ pfu/ml) compared to Large isolates (3.3 ± 1.5 × 10^7^ pfu/ml) at 2 days post-infection (Fig. 3C). However, cytotoxicity did not show significant differences between the two isolates throughout the culture period (Fig. 3D).

### In Vivo Characterization of K15P38 Variants

**Fig. 4:**
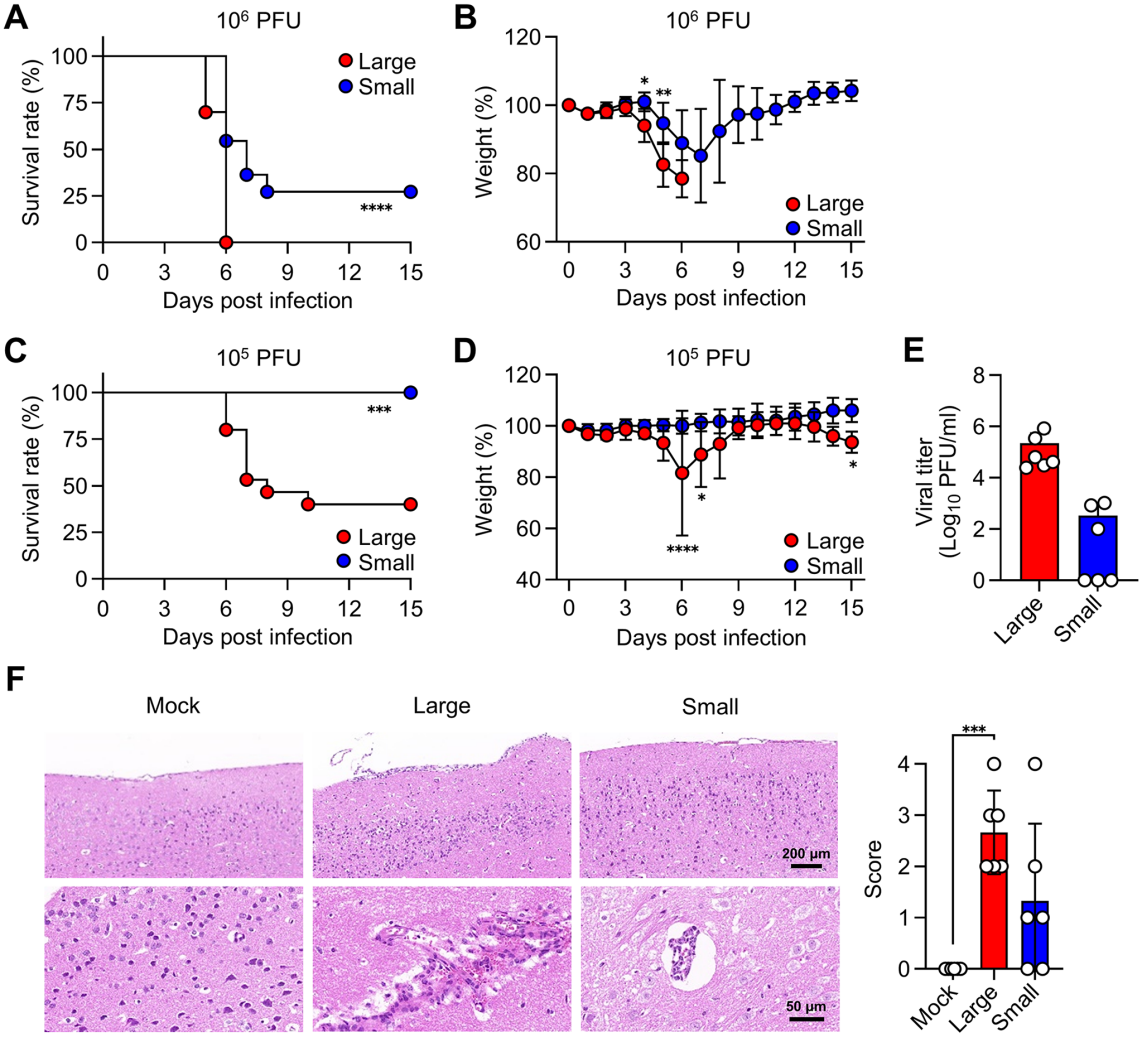
In vivo characterization of K15P38 variants. (**A-D**) BALB/c mice were intravenously infected with 10^5^ (**A, B**) or 10^6^ (**C, D**) pfu of either Large or Small virus (*n* = 10-15). Survival rate (**A, C**) and body weight (**B, D**) of the infected mice were monitored for 15 days post-infection. (**E, F**) Viral titers (**E**) and histopathological scores (**F**) were measured in the brains of mice infected with 106 pfu of either Large or Small virus at 4 days post-infection. Data represent mean ± SD values pooled from two independent experiments (*n* = 6). *p < 0.05; **p < 0.01; ***p < 0.001; ****p < 0.0001.

To investigate the virulence of isolates in vivo, five-week-old female BALB/c mice were infected intravenously with 10^6^ pfu of either Large or Small virus. While all mice infected with Large virus succumbed to death by 6 days post-infection, 27.3% of mice infected with Small virus survived (Fig. 4A). Differences in body weight between the two groups appeared from day 4, and survivors infected with the Small virus began to regain weight from day 7 (Fig. 4B). When a lower dose (10^5^ pfu) of either Large or Small virus was used, all mice infected with the Small virus survived, while only 40% of mice infected with the Large virus survived (Fig. 4C). The body weight of Large virus-infected mice dropped to its lowest at day 6, while no significant weight loss was observed in Small virus-infected mice (Fig. 4D). In accordance with weight loss and survival rates, mice infected with the Large virus exhibited a higher viral burden in the brain on day 4 (Fig. 4E). Furthermore, more severe tissue pathology was observed in cases of Large virus infection (2.67 ± 0.82) compared to those with the Small virus (1.33 ± 1.51), although the difference did not reach statistical significance (Fig. 4F).

**Fig. 5:**
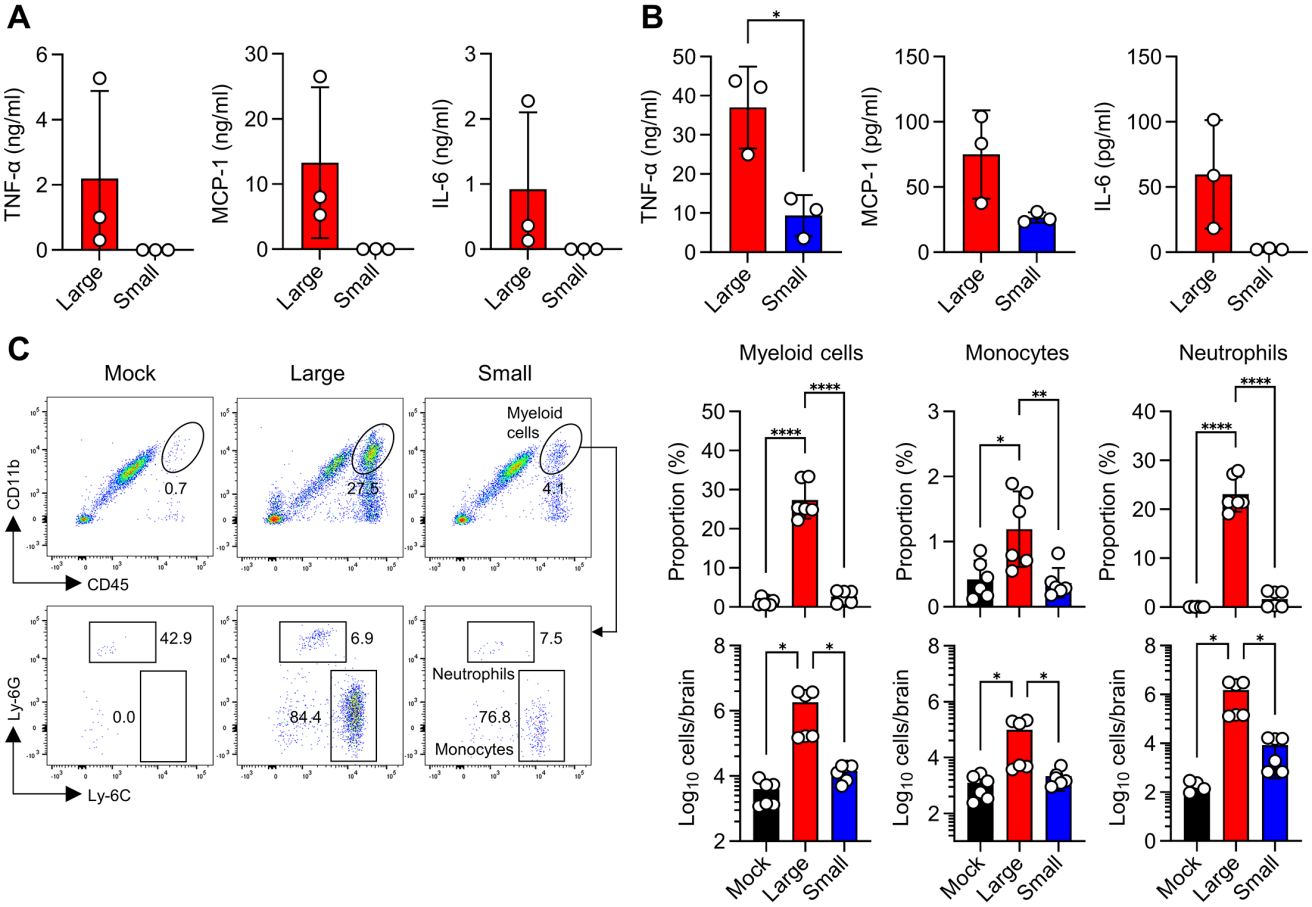
Immunopathogenicity of K15P37 variants. BALB/c were intravenously infected with 10^6^ pfu of either Large or Small virus and sacrificed at 4 days post-infection (*n* = 6). (**A, B**) The levels of TNF-α, MCP-1, and IL-6 were determined in brain (**A**) and plasma (**B**) samples. (**C**) Representative flow cytometry plot and analysis of myeloid cell infiltration, including monocytes and neutrophils, in the brain. Data represent mean ± SD. *p < 0.05, **p < 0.01, ****p < 0.0001.

Next, we examined the immunopathology in mice infected with K15P38 variants. Although there were individual variations, expression of neuropathic cytokines such as TNF-α, MCP-1, and IL-6 was observed in the brains infected with the Large virus, whereas almost no cytokine detection occurred in cases of Small virus infection (Fig. 5A). In the peripheral blood, TNF-α and MCP-1 were detected in mice infected with the Small virus, although their expression levels were relatively lower than in mice infected with the Large virus (Fig. 5B). Myeloid cell infiltration in the brain exhibited a pattern similar to that of the cytokines. Mice infected with the Large virus showed significantly higher myeloid cell (CD45^high^CD11b^+^) infiltration compared to those infected with the Small virus, with these myeloid cells predominantly comprising monocytes and neutrophils (Fig. 5C). Small virus infection did not show a statistically significant increase in immune cell infiltration compared to mock-infected mice. Overall, the differences between the Large and Small isolates were more pronounced in vivo than in vitro, demonstrating an overall attenuated phenotype.

## Discussion

Viruses with RNA genomes, including JEV, inherently possess higher mutation rates compared to those with DNA genomes [20]. Examining the mutant characteristics of circulating viruses is crucial for understanding the diseases they cause, as these mutations enable the virus to interact continuously with the host. The importance of studying the GV genotype of JEV in ROK stems from its unprecedented dominance in detections, which coincides with the timing of increases in patient cases [11, 21-23]. However, despite its significance, K15P38 is the only clinical isolate of GV JEV available for research in the ROK. Therefore, securing and studying variants of this virus is critical for understanding the epidemiology of JE locally and potentially reducing the impact of this virus in other endemic areas.

The large plaque-forming virus isolated from NCCP 43279 had an amino acid sequence that was 100% identical to that previously reported for K15P38. However, the small plaque-forming virus exhibited two amino acid substitutions, found in the PrM and NS5 proteins, respectively. While the E protein primarily plays a crucial role related to antigenicity [24-26], recent studies have demonstrated that specific amino acid substitutions in the non-structural (NS) proteins of JEV significantly influence the growth, virulence, neurovirulence, and replication of the GIII strains [27-29]. Moreover, mutations in the PrM domain have been found to be involved in the attenuation of virulence in the GIII strains [30, 31]. However, mutation studies on the GV genotype have not been extensively conducted due to the scarcity of isolated strains, highlighting the need for further research on virus mutations in GV strains as well.

The PrM protein of JEV plays a pivotal role in both the formation of the virus capsid and the regulation of new virus replicates being secreted from cells [32, 33]. If the PrM protein is modified or defective, the virus capsid formation does not occur properly, leading to a decrease in the virulence of the virus [33]. Recent research has indicated that not only JEV but also the PrM of the Zika virus is involved in pathogenicity in mice [34, 35]. Additionally, the NS5 protein also plays a central role in the replication of viral RNA, and mutations at specific sites of this protein can greatly influence the virus’s replicative ability and pathogenicity [36, 37]. In this study, we reported that amino acid substitutions in the K15P38 variant occurred in two important functional regions. However, further research using reverse genetic technique will be necessary to demonstrate how the mutations we identified in the PrM and NS5 proteins induce small plaque formation and attenuated virulence in mice [38] .

Similarly, the small virus did not exhibit noticeable immunopathology in the brains of infected mice four days post-infection. While we expected mice infected with the small virus to exhibit attenuated pathology due to a lower viral burden compared to those infected with the Large virus, the cytokine levels and myeloid cell infiltration observed were even lower than anticipated. We believe this difference is characteristic of the time point at which the analysis was conducted. We set the time point to check immunopathology on day 4 post-infection because mice infected with 10^5^ pfu of the large virus succumbed to death by this time. However, at this point, mice infected with the small virus had not yet shown significant weight changes. This suggests that the spread of the small virus starts later, and therefore, the pathology observed at this time point reflects a delayed onset characteristic.

Overall, we are reporting on variants derived from K15P38, particularly noting that substitutions in PrM and NS5 significantly influence the in vitro and in vivo characteristics of these variants. While further in-depth studies on more GV JEV isolates are necessary, the findings from this study are expected to be useful for future molecular biological studies of pathogenesis and the development of attenuating vaccine strains.
